# Genome Sequence of *Verrucomicrobium* sp. Strain GAS474, a Novel Bacterium Isolated from Soil

**DOI:** 10.1128/genomeA.01451-17

**Published:** 2018-01-25

**Authors:** Grace Pold, Erin M. Conlon, Marcel Huntemann, Manoj Pillay, Natalia Mikhailova, Dimitrios Stamatis, T. B. K. Reddy, Chris Daum, Nicole Shapiro, Nikos Kyrpides, Tanja Woyke, Kristen M. DeAngelis

**Affiliations:** a Graduate Program in Organismic and Evolutionary Biology, University of Massachusetts–Amherst, Amherst, Massachusetts, USA; b Department of Mathematics and Statistics, University of Massachusetts–Amherst, Amherst, Massachusetts, USA; c U.S. Department of Energy Joint Genome Institute, Walnut Creek, California, USA; d Department of Microbiology, University of Massachusetts–Amherst, Amherst, Massachusetts, USA

## Abstract

*Verrucomicrobium* sp. strain GAS474 was isolated from the mineral soil of a temperate deciduous forest in central Massachusetts. Here, we present the complete genome sequence of this phylogenetically novel organism, which consists of a total of 3,763,444 bp on a single scaffold, with a 65.8% GC content and 3,273 predicted open reading frames.

## GENOME ANNOUNCEMENT

*Verrucomicrobium* sp. strain GAS474 is a novel slow-growing Gram-negative bacterium isolated from the upper mineral soil of a long-term warming experiment (1). Verrucomicrobia represent 23% of the bacteria present in soil (2), but fewer than 50 ecotypes have been cultured and sequenced. Therefore, the characterization and sequencing of members of this group can improve our understanding of vital soil processes, such as carbon cycling.

Soil was collected from the top 10 cm of mineral soil at the Prospect Hill warming experiment at the Harvard Forest Long Term Ecological Research site (42.54°N, 72.18°W) and subsequently dispersed for 30 min in a phosphate buffer-dithiothreitol-sodium pyrophosphate solution. After settling, the solution was spread onto VL55 medium (3) solidified with gellan gum and amended with 0.05 g/liter xylan from birchwood. Plates were incubated aerobically at 22 to 25°C, and GAS474 appeared after 8 weeks.

Cells were prepared for sequencing by growing on an R2A plate incubated for 6 weeks under aerobic conditions at 22 to 25°C; genomic DNA (gDNA) was extracted using the standard Qiagen Genomic-tip protocol for bacteria. Whole-genome sequencing was completed at the Joint Genome Institute on the PacBio RS platform (4). The reads were assembled using the Hierarchical Genome Assembly Process (HGAP) version 2.3.0 (5), leading to a single contig 3.763 Mb in size with 178× coverage.

Gene prediction and functional annotation were performed using the U.S. Department of Energy Joint Genome Institute annotation pipeline (6). The genome contains 3,273 predicted coding sequences, of which, 73.0% were assigned a function and 9.4% were predicted to contain signal peptides using SignalP (7). The genome contains 47 tRNAs and a single rRNA operon. The genome is available for comparative analysis through the Integrated Microbial Genomes (IMG) data management system (8).

GAS474 is phylogenetically and phenotypically distinct from its closest cultured relatives, sharing less than 88% 16S rRNA identity and less than 73% average nucleotide identity with *Methylacidimicrobium*. *fagopyrum*, *Methylacidimicrobium tartarophylax*, *Methylacidimicrobium cyclopophantes*, *Methylacidiphilum fumariolicum* SolV, and *Methylacidiphilum kamchatkense* Kam1. GAS474 lacks the particulate methane monooxygenase and RuBisCO genes that enable its closest relatives to survive as autotrophic aerobic methanotrophs (9, 10). Instead, its genome shows enrichment in genes for secondary metabolite processing (Clusters of Orthologous Groups [COG] category Q; 1.5× increase in fraction of protein-coding reads compared to four closest relatives), pilus formation (COG category W; 5.3× greater), signal transduction (COG category T; 2× greater), and motility (COG category N; 6× greater). GAS474 also shows an expanded ability to process diverse saccharides, having more than twice the fraction (8.5 versus 4.1%, determined using dbCAN [11]) and diversity (80 versus 38 distinct carbohydrate-active enzyme families, and 29 versus 12 glycoside hydrolase families) of genes and the potential to process a broader range of substrates than the primarily peptidoglycan- and α-glycosidic limited abilities (GH113 and GH57) of its methanotrophic relatives. We therefore introduce the genome of *Verrucomicrobium* sp. strain GAS474 as a representative of a novel cultivated lineage in the verrucomicrobial phylogenetic tree.

### Accession number(s).

This whole-genome shotgun project has been deposited in GenBank under the accession no. LT629781. The version described in this paper is the first version, LT629781.1.
